# Adulteration of Cream of Tartar

**Published:** 1849-05

**Authors:** James Grant


*Adulteration of Cream of Tartar.* By Mr. James Grant. —I beg through the medium of the Pharmaceutical Journal to call the attentian of Chemists and Druggists to the adulteration of powdered Cream of Tartar, as it is, I believe, one of those articles which, from its cheapness, &c. is generally supposed not to be worth sophisticating. A specimen obtained from one of the most respectable wholesale druggists in London yielded, in 100 grains, 8½ grains of sulphate of lime (gypsum.)

As cream of tartar is the source to which the chemical student is generally referred for pure carbonate of potash, the exposure of this fraud becomes doubly important.—*Ibid.*

